# More frequent and widespread persistent compound drought and heat event observed in China

**DOI:** 10.1038/s41598-020-71312-3

**Published:** 2020-09-03

**Authors:** Rong Yu, Panmao Zhai

**Affiliations:** State Key Laboratory of Severe Weather, Chinese Academy of Meteorological Sciences, Beijing, 100081 China

**Keywords:** Climate sciences, Natural hazards

## Abstract

Compound drought and heat event (CDHE) causes severe impacts on agriculture, ecosystem, and human health. Based on daily maximum surface air temperature and meteorological drought composite index data in China, changing features of CDHEs in warm season from 1961 to 2018 is explored at a daily time scale based on a strict and objective definition in this study. Results reveal that CDHEs have occurred more frequently and widely in China, especially since the late 1990s. Notably, such changes are more obvious in Southwest China, eastern Northwest China, northern North China, and the coastal area of southeastern China. A prominent feature is that persistent CDHEs on a daily scale have increased significantly. To better understand climate change of compound extreme events, further studies on the physical mechanism, especially attribution analyses at a regional scale, are urgently needed.

## Introduction

Extreme weather and climate events have been widely studied over the past decades^1–3^. Research on changes in extreme heat events and drought events has been conducted in different regions of the world^4,5^. In China, frequency, intensity, and affected areas of extreme heat events show increase trends in most areas^6,7^. Drought has enhanced in North China and Southwest China during the past decades^8,9^. Further studies shown that extreme temperature and drought events have happened more frequently and widely under global warming, and they are projected to further enhance with temperature rising^10,11^. Recently, concurrent drought and heat events seen in 2018 Europe and 2019 Australia have attracted the attention of science community^12^. Such types of compound extreme events induce aggravated impacts on agriculture, ecosystem, water security, and human health^13–16^.

Studies are emerging in compound drought and heat events (CDHEs). As documented, Europe has been widely and frequently threatened by CDHEs in recent years^13,17,18^. In Africa, the increasing trend has been observed and projected for summer drought and heat waves^15,19^. In the United States, a substantial increase in CDHEs has been highlighted^20,21^. Meanwhile, exacerbated CDHEs have been investigated in some regions from different perspectives in China^22–24^.

However, in most previous studies, monthly drought indices were used as a background to explore the changes in CDHEs^13,22,25,26^. Following such approaches, the duration of CDHEs could not be accurately provided to some extent, since daily features based on most previous studies have been obscured. For some other studies^23,27^, dry conditions were used to analyze changes in CDHEs. In that case, those detected and attributed changes are not related to drought directly. As a matter of fact, a CDHE which has mixed impacts of heat weather and drought conditions persisting for several days or weeks can induce serious impacts. Also, the detection and attribution of CDHEs, and feedback between extreme drought and heat events, are very important topics, which require a better index to understand the changing features^17,28,29^.

In this study, meteorological drought composite index (MCI), a daily drought index used in China’s drought monitoring^30,31^, is applied to derive CDHEs in this study. Considering that changes in drought and heat events bring about a disastrous impacts on agriculture, ecosystem, and human health, spatial–temporal changes in daily CDHEs during warm season (from May to October) of 1961–2018 in China are investigated. Besides, intensity, frequency, persistence, and spatial distribution are important properties for understanding extreme event changes^32,33^. Based on the most updated datasets and the newly defined CDHE index, these changes in the above mentioned properties of CDHEs in China are also studied and reflected in this investigation.

## Changes in compound drought and heat events

**Figure 1:**
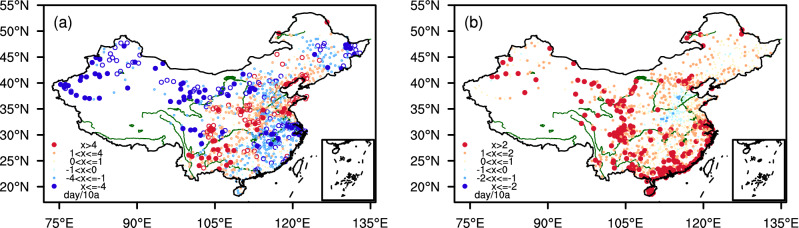
Spatial distribution of trends in the number of drought days (**a**) and trends of heat days (**b**) in the warm season of 1961–2018 in China. Statistical significance is detected using Mann–Kendall non-parametric test and the linear trend is quantified using Theil-Sen trend estimation (filled dots refer to stations with statistical significance at 90% confidence level). Maps are generated using NCAR Command Language (The NCAR Command Language (Version 6.6.2) [Software]. (2019). Boulder, Colorado: UCAR/NCAR/CISL/TDD. https://www.ncl.ucar.edu/).

Based on the long-term time series of daily MCI and daily maximum surface air temperature, the trends in drought (Fig. 1a) and heat events days (Fig. 1b) are separately analyzed firstly. Results indicate that number of drought days illustrates a decreasing trend in southeastern China and Northwest China from 1961 to 2018, while it shows an increasing trend in Southwest China and North China (Fig. 1a). Such a changing pattern is generally consistent with that the conclusion on changes in precipitation by Zhai et al.^34^. As for Southwest China and North China, dry-spell has prevailed in recent years, thus triggered drought events frequently happened^33^.

For the heat events, Fig. 1b reveals that the number of heat events days shows a widespread increasing trend in China. Different from drought, it has increased significantly in Northwest China, southeastern China, South China, and the Yangtze River Valley. However, there exists a negative trend in the number of heat event days in southern North China, where the downtrend is possibly reflected as a regional response to inter-decadal and inter-annual variability^35,36^. Also, some other studies suggested that such a unique regional changing feature under global warming is possibly attributed to changes in aerosol, and regional land use and management^1^.

**Figure 2:**
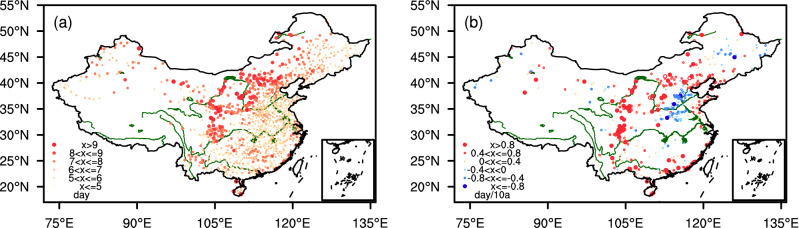
Distribution of the climate average and trend of CDHE days in the warm season during 1961–2018 ((**a**) average of all compound days for the period of 1961–2018; (**b**) trend of all CDHE days. Statistic significance is detected using Mann–Kendall non-parametric test and the linear trend is quantified using Theil-Sen trend estimation(Filled dots stand for the station with statistical significance at 90% confidence level)). Maps are generated using NCAR Command Language (The NCAR Command Language (Version 6.6.2) [Software]. (2019). Boulder, Colorado: UCAR/NCAR/CISL/TDD. https://www.ncl.ucar.edu/).

As for drought and heat events, Fig. 2a displays the spatial distribution of the average number of CDHE days in the warm season. As illustrated, CDHEs increase in frequency from south to north in China. Notably, the average increase is more than 10 days in northern North China, Northwest China, and Southwest China. It is more frequent in the arid or semi-arid climate zones. Figure 2b provides the trend distribution for the number of CDHE days. The results indicate that the number of CDHE days has increased in most parts of China with a trend of more than 0.8 day/10a. However, it has decreased in southern North China and some eastern parts of Northeast China. Similar evidence is also identified by some regional research studies in China^37–39^.

To further understand why some regions in China show decrease trends in CDHEs, we have compared with evidence shown in Fig. 1a,b. Obviously, the decreasing trend in the number of CDHE days in southern North China is mainly because of decrease in the number of heat event days, that in the eastern part of Northeast China and the Middle and Lower Reaches of the Yangtze River is mainly due to increase in precipitation. Nevertheless, China has experienced more frequent and widespread CDHEs in general.

**Figure 3:**
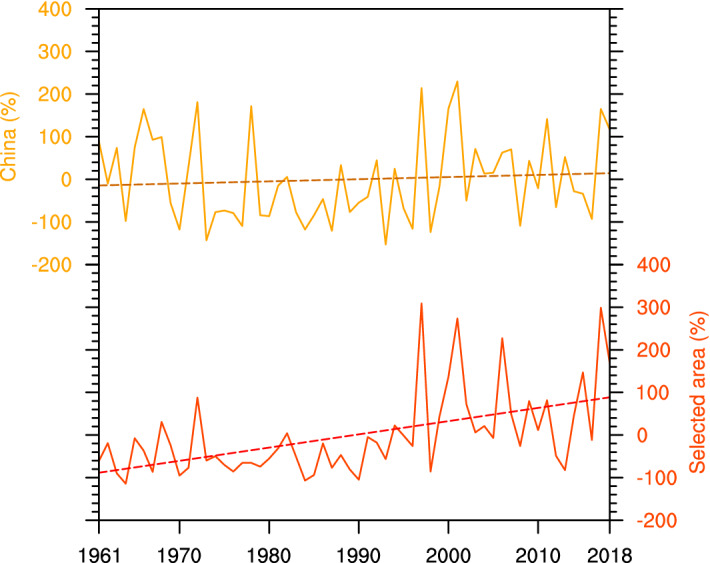
Normalized time series of the annual number of CDHE days in China (brown line) and in the selected area (stations with increase trends in CDHE days, mainly in Southwest China, eastern Northwest China, northern North China and coastal area of southeastern China, red line) in warm season during 1961–2018, respectively. The map is generated using NCAR Command Language (The NCAR Command Language (Version 6.6.2) [Software]. (2019). Boulder, Colorado: UCAR/NCAR/CISL/TDD. https://www.ncl.ucar.edu/).

Additionally, the time series of the annual number of CDHE days in the warm season is shown in Fig. 3. For China as a whole, the frequency of CDHEs has increased with a trend of 5%/10a, indicating that CDHEs has happened more frequently. The occurrence of CDHEs is relatively lower during the early 1970s to mid-1990s, but it has increased quickly since the late 1990s. Regionally, as shown in Fig. 2b, the increased frequency of CDHEs is mainly found in Southwest China, eastern Northwest China, northern North China, and the coastal area of southeastern China. To further investigate regional change features, time series in the number of CDHE days for the stations with an identified positive trend in Fig. 2b (regard as selected area) is established. Results show that the trend in the occurrence of CDHEs in such stations is about 31%/10a, which is 5-time larger than the whole China average. Moreover, a greater increase in CDHEs frequency is observed since the late 1990s with the top 10 highest frequencies mostly in the twenty-first century. It indicates that compound events happen more frequently and significantly in Southwest China, eastern Northwest China, northern North China, and the coastal area of southeastern China, especially since the late 1990s.

**Figure 4:**
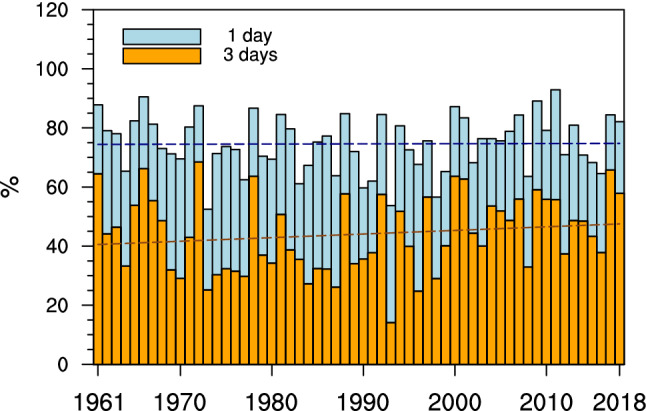
Time series of the spatial extension of CDHEs and persistent CDHEs in China in warm season during the time period of 1961–2018 (Lines refer to linear regression, blue and orange bars stand for CDHEs and persistent CDHEs, respectively). The map is generated using NCAR Command Language (The NCAR Command Language (Version 6.6.2) [Software]. (2019). Boulder, Colorado: UCAR/NCAR/CISL/TDD. https://www.ncl.ucar.edu/).

To further analyze the change in spatial coverage of CDHEs, the time series of spatial area for CDHEs of different duration are provided in Fig. 4. As shown, the spatial extension of CDHEs is smaller than normal in the 1970s to mid-1990s, but larger in 1960s and late 1990s to 2010s. There is a non-significant increase trend (0.1%/10a) in spatial area of CDHEs. However, the increasing trend is significant for persistent CDHEs. Specifically, the trend of the area coverage for persistent CDHEs is about 1.2%/10a in China from 1961 to 2018. It implies that persistent CDHEs are occurring in more regions in China. Based on Fig. 3 and 4, it can be concluded that CDHEs have happened more widely and persistently in China, especially since the late 1990s.

As supported by the the previous studies^40^, the change in CDHE frequency and spatial extension before and after the late 1990s is likely related to the significant increase in both heat events and drought events in China since the late 1990s. The possible causes of the increase in China’s heat events and drought events have been explained to be related to anthropogenic global warming and internal variabilities, such as the Pacific Decadal Oscillation and Atlantic Multidecadal Oscillation^41–44^.

**Figure 5:**
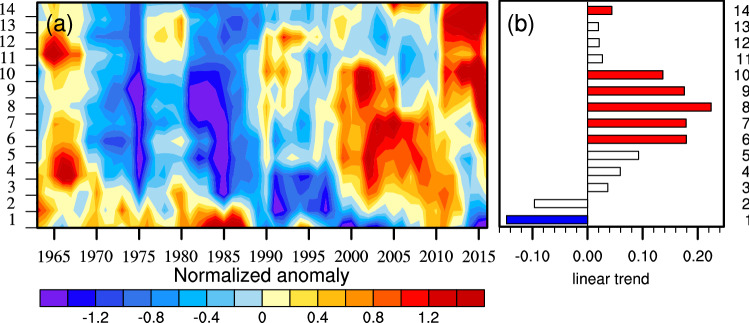
(**a**) temporal evolution of normalized anomaly of the fractional contribution of CDHEs with different duration to the total number of CDHE days for stations in China in warm season smoothed with a 5-year running mean, and (**b**) trend (%/10a) in the normalized anomaly of the fractional contribution of CDHEs with different duration during the period of 1961–2018 (Filled bars stand for the station with statistical significance at 90% confidence level). Maps are generated using NCAR Command Language (The NCAR Command Language (Version 6.6.2) [Software]. (2019). Boulder, Colorado: UCAR/NCAR/CISL/TDD. https://www.ncl.ucar.edu/).

Furthermore, the duration structure of CDHEs has changed since the late 1990s (Fig. 5). The change in the fractional contribution of non-persistent CDHEs shows more than normal before the 1990s (Fig. 5a). However, it has become less thereafter. Meanwhile, the fractional contribution of persistent CDHEs is less than normal before the 1990s and then it has increased since late 1990. As it is shown in Fig. 5b, the increasing trends for persistent CDHEs between 6 and 10 days are significant from 1961 to 2018. It can be concluded that fractional contribution has decreased for non-persistent CDHEs, while it has increased for persistent CDHEs. In general, CDHEs in China tend to happen more persistently in the warm season from 1961 to 2018.

## Conclusion and discussion

In this study, a daily index reflecting the compound drought and heat event (CDHE) has been defined for the warm season based on the most updated daily temperature and drought index used operationally in China. This is different from the past studies which were based on a longer timescale drought background. It allows to more objectively reflect the changes in CDHEs and their persistence at daily timescale. Results reveal that CDHEs in China have become more frequent and more widespread, especially since the late 1990s. The increasing trend in the frequency of CDHEs in China is 5%/10a. We have also seen distinct regional features in the change of CDHEs in China as the increased frequency of CDHEs is more obvious in Southwest China, eastern Northwest China, northern North China, and coastal area of southeastern China, with a trend about 31%/10a.

Such changing feature seems to link to changes in both heat events and drought events. Generally, either heat events or drought events in China has been increased since the late 1990s, possibly due to the combined effects of long term trend of global warming and multi-decadal/multi-annual variability (e.g. Pacific Decadal Oscillation, Atlantic Multidecadal Oscillation, and El Nino)^42,43,45^. As a result, CDHEs have increased spatially and temporally.

Furthermore, CDHEs are found to have become more persistent. As CDHE is a concurrent event of drought and heat, some studies have already found that both heat and drought events in China have been prolonged during the past decade^43,46^ mainly because of global warming, the influence of ocean conditions, and regional land-use and land management changes. Noticeably, there may exist positive feedback between heat events and drought events^47,48^, especially under the global warming background. Self-intensification and self-propagation of compound events will exist with the aid of local and teleconnection feedback^49^. Possibly due to such reasons, CDHEs tend to happen more persistently under the background of increased drought and heat events since the late 1990s. Besides, soil moisture deficits before the onset of concurrent heat and dry extreme may also play an important role^17^. Understanding of these mechanisms will greatly support explain why CDHEs have increased and prolonged in China, especially in arid and semi-arid regions in China.

However, the interplay of multi-time scale variability and anthropogenic forcing on CDHEs is a complicated issue. Attribution study for change in compound extreme events such as CDHEs is still a challenging research topic. Meanwhile, a comprehensive understanding of the feedback between heat events and drought events under the circumstance of CDHEs in China is insufficient and unclear at present. All these related studies in the future will improve the understanding of change in CDHEs at a regional scale and support climate change projection for extreme events, especially compound extreme events.

## Data and methods

### Data and definition

A long-term daily surface air temperature dataset consisting of 2,419 stations from 1961 to 2018 is obtained from China National Meteorological Information Center (NMIC). This dataset has been conducted rigorous quality control^50^. It has been widely used in the previous studies^51,52^. Additionally, an established drought index, the MCI dataset of 2,423 stations from 1961 to 2018 is provided by the National Climate Center of China. Different from some widely used drought index, such as standardized precipitation index (SPI)^53^ and Palmer drought severity index (PDSI)^54^, MCI provides a more objective drought index of daily scale and has been operationally used for drought monitoring in China for over 10 years^30,31^.

Furthermore, some stations are not taken into account if: (1) they do not cover for the whole period from 1961 to 2018, (2) stations have experienced relocation with their horizontal change beyond 20 km or vertical change over 50m^33^, (3) total percentage of missing data during the period is over 5%, (4) stations only include temperature data or MCI data. After those strict procedures, 1,250 stations are selected for this study. Based on MCI and maximum surface air temperature from 1961 to 2018, CDHE days are defined objectively as concurrent events of heat days and drought days (Related definitions are illustrated in Table 1).

**Table 1:** Summary of related definitions for compound drought and heat event.

Event	Definition
Drought day	Light drought : − 1.0 < *MCI* < − 0.5 Moderate drought: − 1.5 < *MCI* ≤ − 1.0 Severe drought: − 2.0 < *MCI* ≤ − 1.5 Extreme drought: *MCI* ≤ − 2.0
Heat day	Tmax > Tmax_95_ Tmax_95_ refer to 95 percentile of daily maximum air temperature in the warm season of 1961–1990
Compound drought and heat event (CDHE)	*MCI* ≤ − 0.5 and Tmax ≥ Tmax_95_
K-day compound drought and heat event	a K-day CDHE is defined if the CDHE persists for *K* days, then the CDHE terminates when *MCI *> − 0.5 or Tmax < Tmax_95_
Non-persistent compound drought and heat event	CDHE with a duration of 1- and 2-days
Persistent compound drought and heat event	CDHE with a duration of longer than 2 days

Here, to derive a universal definition and to easily conduct spatial comparison for entire China, 95 percentile of daily maximum air temperature (Tmax_95_) in the warm season of 1961–1990 is used to define a heat day event. Based on this threshold, Tmax_95_ in about 90% stations is higher than 30 °C and in about 45% stations over 35 °C. Only in few stations in the places over 3000 km elevation of the Tibetan Plateau region, Tmax_95_ is slightly below 25 °C. Nevertheless, such threshold has been also used to define high temperatures for a heat event locally^55^. Also, the percentile threshold definition for heat day is used in many recent studies related to compound event^28,56^.

### Methods

To analyze the persistence change of the compound event, CDHEs are divided into 14 categories based on the number of persistent days from 1 to 14 days. CDHE lasting for more than 14 days is allocated into the category of 14-day CDHE. Additionally, for a given category *K*, the fractional contribution is calculated as the ratio of the total number of *K*-day CDHEs to the total number of CDHEs.

Following Zolina et al.^32^, normalized anomalies of the fractional contribution of CDHEs with different duration to the total number of CDHE days are calculated, derived from all selected stations in the warm season and smoothed with a 5-year running mean. If the fractional contribution of CDHEs with longer (shorter) duration is found to increase, it means that CDHEs tend to happen in a more persistent (non-persistent) way.

Additionally, the in situ observation network is dense in eastern China but scarce in western China. To avoid spatial sampling bias in whole country statistics, the percentage of the spatial coverage of CDHEs is calculated as a ratio of areal coverage of CDHEs to the valid observation area in China based on averages inside 1° × 1°grids. The same approach also applied for persistent CDHEs’s change study.
